# Effects of Systemic Metabolic Fuels on Glucose and Lactate Levels in the Brain Extracellular Compartment of the Mouse

**DOI:** 10.3389/fnins.2017.00007

**Published:** 2017-01-19

**Authors:** Alexandria Béland-Millar, Jeremy Larcher, Justine Courtemanche, Tina Yuan, Claude Messier

**Affiliations:** School of Psychology, University of OttawaOttawa, ON, Canada

**Keywords:** brain metabolism, blood-brain barrier, galactose, insulin, pyruvate, beta-hydroxybutyrate, fructose, astrocytes

## Abstract

Classic neuroenergetic research has emphasized the role of glucose, its transport and its metabolism in sustaining normal neural function leading to the textbook statement that it is the necessary and sole metabolic fuel of the mammalian brain. New evidence, including the Astrocyte-to-Neuron Lactate Shuttle hypothesis, suggests that the brain can use other metabolic substrates. To further study that possibility, we examined the effect of intraperitoneally administered metabolic fuels (glucose, fructose, lactate, pyruvate, ß-hydroxybutyrate, and galactose), and insulin, on blood, and extracellular brain levels of glucose and lactate in the adult male CD1 mouse. Primary motor cortex extracellular levels of glucose and lactate were monitored in freely moving mice with the use of electrochemical electrodes. Blood concentration of these same metabolites were obtained by tail vein sampling and measured with glucose and lactate meters. Blood and extracellular fluctuations of glucose and lactate were monitored for a 2-h period. We found that the systemic injections of glucose, fructose, lactate, pyruvate, and ß-hydroxybutyrate increased blood lactate levels. Apart for a small transitory rise in brain extracellular lactate levels, the main effect of the systemic injection of glucose, fructose, lactate, pyruvate, and ß-hydroxybutyrate was an increase in brain extracellular glucose levels. Systemic galactose injections produced a small rise in blood glucose and lactate but almost no change in brain extracellular lactate and glucose. Systemic insulin injections led to a decrease in blood glucose and a small rise in blood lactate; however brain extracellular glucose and lactate monotonically decreased at the same rate. Our results support the concept that the brain is able to use alternative fuels and the current experiments suggest some of the mechanisms involved.

## Introduction

The study of mammalian brain bioenergetics has centered on the transport of glucose and its metabolism in the presence of oxygen. This view is based on the inescapable fact that the brain eventually ceases to function in the complete absence of either glucose or oxygen. The various aspects of the brain's metabolism of glucose have been extensively studied and form the basis of our understanding regarding how the brain uses energy. The possibility that the brain could use different metabolic substrates or that intermediary metabolic substrates could play a major role in brain metabolism has been suggested at various times in the history of brain metabolism research (Schurr, 2014). The revival of some earlier hypotheses have crystalized around the astrocyte-lactate shuttle hypothesis. Modeled from the lactate shuttle in muscles (Brooks, 1986, 2000; Brooks et al., 1999; Giugliano et al., 2008), the Astrocyte-to-Neuron Lactate Shuttle (ANLS) hypothesis was first proposed by Pellerin and Magistretti (1994). This hypothesis postulates that in times of increased neuronal activity, and thus energy demand, astrocytes take up blood glucose via their particularly well-positioned endfeet on capillaries and convert this glucose to lactate. Lactate is then shuttled to surrounding neurons for oxidative phosphorylation, avoiding the glycolytic steps involved in the full metabolism of glucose.

Support for the ANLS hypothesis comes from a variety of research fields. Some key findings center on molecular studies that have identified a mitochondrial redox complex comprised of a monocarboxylate transporter (MCT1) and lactate dehydrogenase (LDH) enzyme in neurons (Brooks et al., 1999; Hashimoto and Brooks, 2008; Hashimoto et al., 2008; Passarella et al., 2008). Consequently, neuronal mitochondria would appear to possess the ability to take in lactate and convert it to pyruvate in order to sustain oxidative metabolism. Secondly, studies relating to memory performance in chicks (Baker and Edwards, 2007; Gibbs and Hertz, 2008) and rats (Newman et al., 2011) demonstrate the importance of lactate transfer for memory consolidation. Lastly, *in vitro* rat hippocampal slices prove to be quite efficient at metabolizing lactate (Schurr et al., 1988, 1997; Brooks, 2007). For a more complete presentation of the evidence supporting this hypothesis, readers are directed to the following reviews (Magistretti and Pellerin, 1996; Pellerin et al., 1998, 2007; Schurr et al., 1999; Schurr, 2006; Pellerin, 2008; Bouzier-Sore and Pellerin, 2013). Additionally, for a review of the similarities between the muscular lactate shuttle and the cerebral ANLS hypothesis, see Brooks (2002, 2009) and Gladden (2004).

A competing hypothesis, the neuron-to-astrocyte lactate shuttle (NALS) (Mangia et al., 2009), is based on a number of arguments that center on the different characteristics of GLUT1, the glucose transporter found on the epithelial cells of blood vessels and on astrocytes, and GLUT3, the transporter found mainly on the neuropil of neurons (Chih et al., 2001; Chih and Roberts, 2003; Mangia et al., 2009, 2011; Massucci et al., 2013). GLUT1 has a high affinity for glucose (Heyes, 2010) and is also capable of transporting other hexose at a much-reduced rate (Gould et al., 1991). GLUT1 appears to be more prevalent on the luminal membrane of the vascular epithelium compared to the abluminal side (Farrell and Pardridge, 1991; Simpson et al., 2001; Cornford and Hyman, 2005; Kubo et al., 2015), though the exact ratio and distribution is debated (Farrell and Pardridge, 1991; Akiyoshi et al., 1999). This asymmetrical distribution of GLUT1 and the presence of hexokinase within the cells suggest that glucose is mostly transferred unidirectionally from blood to brain even though, given greater brain glucose concentrations, glucose could be transported from brain to blood. Based on its transport kinetics, the ability the transporter GLUT3 to transfer glucose is thought to be greater than GLUT1, making glucose entry into neurons more efficient (Simpson et al., 2007, 2008). However, the necessity for glucose to diffuse through endothelial cells, pericytes, astrocytes as well as the extracellular space can limit neuronal access suggesting that high affinity transporters may be essential to utilizing any residual glucose found in the extracellular space following its transport through the numerous membranes.

Similar to glucose, lactate transport between blood and brain appears to be bidirectional and favors the use of the monocarboxylate transporter MCT1. MCT1 transport of lactate begins with a proton binding and ends with the dissociation of the proton and the lactate ion on the opposite side of the membrane (Halestrap and Price, 1999). MCT1 is also capable of transporting other naturally-occurring monocarboxylates, such as pyruvate, lactate, acetoacetate, and ß-hydroxybutyrate. The transport of these other monocarboxylates, however, occurs at a lower rate (Halestrap and Price, 1999; Juel and Halestrap, 1999; Halestrap and Wilson, 2012). In contrast to GLUT1, MCT1 appears to be equally distributed between the luminal and the abluminal membrane of epithelial cells (Leino et al., 1999; Kubo et al., 2015).

Beyond examining the impact of systemically administered nutrients on brain extracellular lactate and glucose, we wanted to revisit the mechanisms involved in the memory-facilitating effects of sugars (Gold, 1995; Messier, 2004). Glucose has been shown to improve memory in rats, mice, pigeons, and humans (Messier and Destrade, 1988; Parsons and Gold, 1992; Messier et al., 1998, 1999; Parkes and White, 2000) while fructose effect has been demonstrated in rats (Messier and White, 1987; Rodriguez et al., 1994). Both systemic and intracerebral injections of glucose improve memory performance (Messier and White, 1987; McNay et al., 2000). Additionally, intra-hippocampal injections of lactate are also capable of improving memory. In contrast, the intra-hippocampal injection of the MCT2 (the predominantly neuronal monocarboxylate transporter) blocker alpha-cyano-4-hydroxycinnamate (4-CIN) impairs memory performance (Newman et al., 2011), hinting toward an important role of lactate transport in memory. As a first step, we examined the effects of the systemic injection of alternative fuels on systemic and extracellular glucose and lactate fluctuations in order to better understand the mechanisms underlying brain energetics.

## Materials and methods

### Animals

Sixty one, 15–20 week old, male CD1 mice (Charles River Canada, St-Constant, Québec, Canada) were individually housed and maintained on a reverse 12-h night/day cycle with light on at 7 p.m. Mice had *ad libitum* access to standard chow (Teklad Global 18% Protein 2018, Teklad Lab Animal Diets, Envigo, Mississauga, Canada) and water, unless otherwise specified. All testing was conducted during the night phase of the cycle with the use of dim red lighting. All procedures in this study adhere to guidelines of the Canadian Council on Animal Care and were approved by the Animal Care Committee of the University of Ottawa.

### Surgical procedure

Pre-surgical treatment included a 0.05 mg/kg subcutaneous (s.c.) injection of buprenorphine hydrochloride (Reckitt Benckiser Healthcare, Hull, North Humberside, UK) as well as 1 ml of 0.9% saline (Hospira, Montreal, Canada).

**Figure 1:**
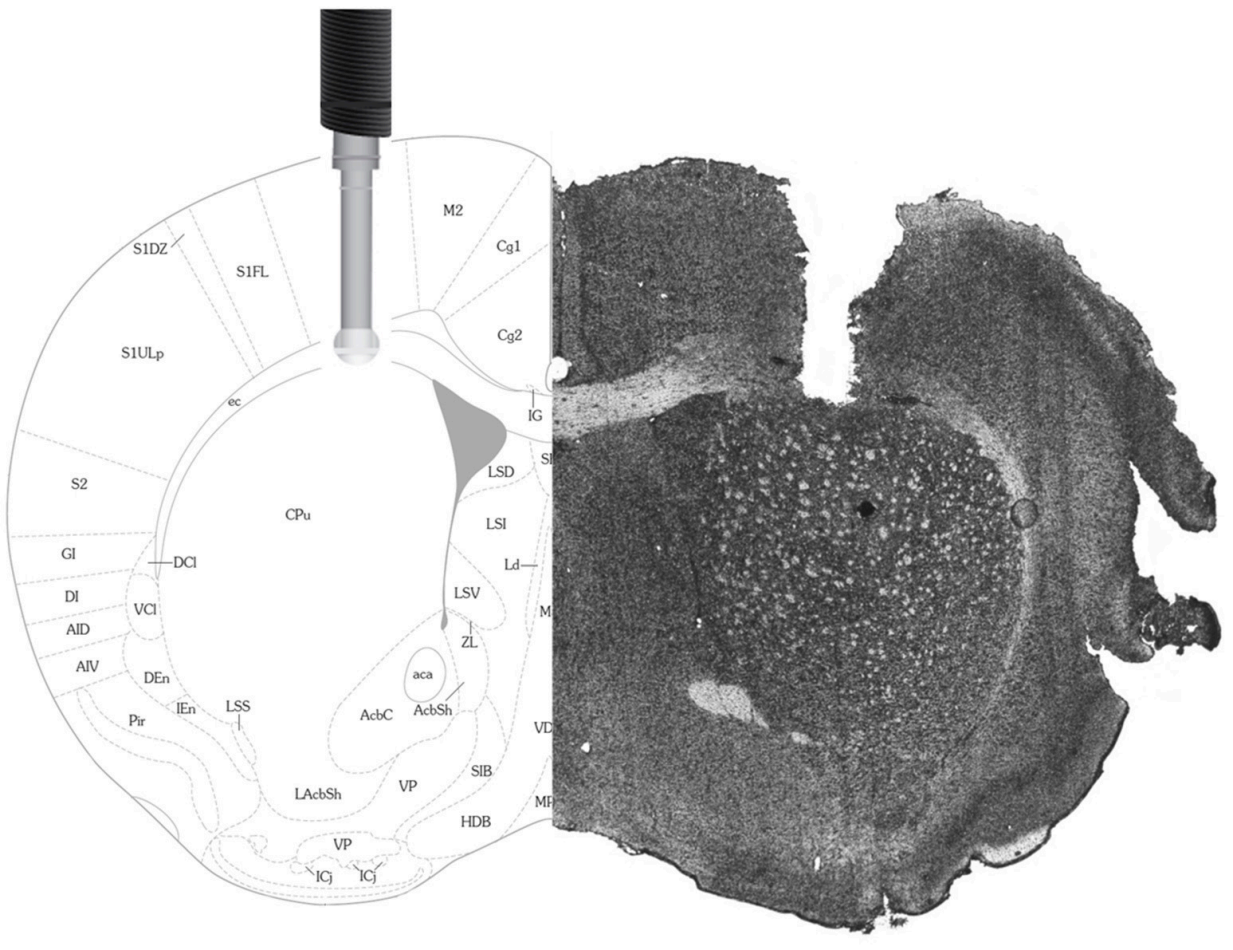
**Histology and Stereotaxic Location of Electrodes**. On the right is a picture of a 14 μm mouse brain slice taken following the implantation of an electrode in the primary motor cortex (M1). For comparison, on the left is a picture from the Franklin and Paxinos (2008) Mouse Brain Atlas used to determine stereotaxic coordinates and confirm electrode placement in the primary motor cortex.

Immediately prior to surgery, mice were anesthetised using 4–5% isoflurane (Fresenius Kabi Canada Ltd., Richmond Hill, ON) then secured in a stereotaxic frame (David Kopf Instruments, Tujunga, CA, USA). Mice were maintained under anesthesia during the surgery with 1–2.5% isoflurane and kept warm with a heated pad (TP650, Gaymar Industries, Orchard Park, NY, USA). Based on the stereotaxic atlas of Franklin and Paxinos (2008), two guide cannulas (BASi cannulas Bioanalytical Systems, West Lafayette, IN) were positioned above the left and right primary motor cortex. The target coordinates, from Bregma, were ± 0.18 mm lateral and +1 mm anterior. The cannulas were positioned just beneath the skulls surface so that the sensing cavity (1 mm) of the electrochemical electrode (inserted later, immediately prior to testing) would be situated within the primary motor cortex of the mouse (Figure 1). The guide cannulas were fixed using a UV-polymerized compound (CG3 & CG4, Clear Cure Goo, Southlake, TX, USA) and four 0.10″-inch screws positioned anterior and posterior to the two guide cannulas.

Post-surgical analgesia included application of transdermal bupivacaine (Chiron Compounding Pharmacy Inc., Guelph, ON, Canada) and s.c. injections of buprenorphine hydrochloride twice daily for 3 days. Mice were given 7–8 days to recover before testing.

### Electrodes

The use and details of these commercially available electrodes have been extensively detailed elsewhere (Wilson and Gifford, 2005; Wakabayashi and Kiyatkin, 2012; Kiyatkin et al., 2013; Wakabayashi et al., 2015). Briefly, extracellular brain levels of glucose and lactate were monitored using two platinum enzyme-linked electrodes (7005-Glucose-C and 7005-Lactate-C; Pinnacle Technology Inc., Lawrence, KS, USA). Sensors were prepared from Pt-Ir wire of 180 um diameter, wrapped concentrically with an AgCl reference electrode. Near the tip of the electrode, a 1 mm section is coated with either glucose or lactate oxidase. These enzymes oxidize the analytes of interest, resulting in an equivalent release of hydrogen peroxide (H_2_O_2_). H_2_O_2_ is subsequently detected and recorded as an amperometric oxidation current by the electrode (Hu and Wilson, 1997). Data was collected at 1 Hz, transmitted from a potentiostat to a computer and recorded with the help of Sirenia Acquisition Software (Pinnacle Technology Inc., Lawrence, KS, USA). In addition to the oxidase enzymes, the metabolite sensitive surface is also coated with a series of membranes to increase the specificity and selectivity of the electrodes. For example, the potential contribution of ascorbic acid is reduced with the presence of ascorbic acid oxidase. These particular enzymes convert the electroactive ascorbate, which has the potential to interfere with the electrodes recordings, to non-electroactive dehydroascorbate and water (Hu et al., 1994).

### Calibration

Immediately prior to and following *in vivo* testing, the electrodes were calibrated according to factory recommendations. Briefly, the electrodes were gently placed in 100 mM PBS (pH 7.4) at 23°C (pre-testing) or 37°C (post-testing) and tested for metabolite sensitivity by adding glucose (1 mM) and lactate (0.1 mM) to the PBS solution, as well as selectivity with the use of a single ascorbic acid (0.25 mM) addition.

Pre-calibration curves demonstrated high sensitivity with incremental linear increases for both glucose (average sensitivity 3.32 nA/1 mM) and lactate (average sensitivity 3.40 nA/0.1 mM) electrodes at 23°C. Both glucose and lactate electrodes also exhibited high levels of selectivity by demonstrating minimal sensitivity to ascorbic acid (average sensitivity 0.18 nA/ 0.25 mM and 0.75 nA/0.25 mM, respectively). The post-calibration curves, obtained at 37°C, demonstrated similar results with linear increases for both glucose (average sensitivity 5.14 nA/1 mM) and lactate (average sensitivity 4.70 nA/ 0.1 mM) electrodes. Post calibration curves for ascorbic acid suggested a certain level of wear on the electrodes, most likely due to the insertion and removal process of these sensitive testing instruments (average sensitivity 0.79 nA/0.1 mM and 4.21 nA/0.1 mM for glucose and lactate, respectively).

### Brain extracellular testing procedure

Following electrode calibration, mice were lightly anesthetised with isoflurane and the electrodes were inserted into the motor cortex through the surgically implanted guide cannulas and attached to the pre-amplifier. Mice were tested in a custom-made square polymethyl methacrylate-testing cage with standard bedding (Teklad 7097 Corncob bedding, Envigo, Mississauga, Canada) and water. A mounted camera (Pinnacle Technologies) above the cage recorded the duration of the testing and was synchronized with the electrode sampling. Mice had free access to water but chow was removed 2 h prior to injection and returned 2 h post-injection.

Approximately 2 h following insertion, when the electrodes returned stable readings, mice received a 2 g/kg intraperitoneal (i.p.) injection of either glucose (Dextrose Anhydrous, EMD Chemicals, Gibbstown, NJ, USA; *n* = 4), lactate (Lactic Acid, Acros Organics, NJ, USA; *n* = 10), fructose (D-Fructose, Fisher Scientific, Fair Lawn, NJ, USA; *n* = 6), pyruvate (Sodium Pyruvate, Fisher Scientific, Fair Lawn, NJ, USA; *n* = 6), galactose (Sigma Aldrich, St. Louis, MO, USA; *n* = 6), ß-hydroxybutyrate (Sigma Aldrich, St. Louis, MO, USA; *n* = 5), or insulin at 1.2 I.U. (Novo Nordisk Canada Inc., Mississauga, ON; *n* = 7). Saline (0.9%; Hospira, Montreal, Canada; *n* = 4) injections were used as a control group. This particular dosage was chosen based on previous results that demonstrated the ability of 2 g/kg of sugar to improve memory (Messier and White, 1987). Additionally, this dosage also corresponds to the amount of sugar these animals readily ingest (Messier and White, 1984). Extracellular data was collected for 2 h post-injection. When the testing was complete, mice were immediately perfused. Animals that were used to test the effects of systemic injections on brain extracellular levels of lactate and glucose were different than those used in subsequent tests on blood glucose and lactate measurements.

### Blood tests

Blood samples were obtained by gently piercing the dorsal tail vein with a 23 gauge needle (Becton, Dickinson and Company, Franklin Lakes, NJ) and massaging the tail for blood extraction. To avoid measuring pooled blood, the first blood drop was wiped before collection. Plasma glucose and lactate concentrations (mmol/L) were measured with a glucose (Accu Chek, Aviva, Roche Diagnostics, Mannheim, Germany) or lactate meter (StatStrip Xpress, Nova Biomedical, Innovation House, Runcorn, Cheshire, UK). Prior to testing, mice were transferred to the testing room within their respective testing cage for 1 h in order to acclimate. A single glucose and lactate baseline measure was taken at the end of the acclimatization period. Afterwards mice received a 2 g/kg i.p. injection of either glucose (*n* = 4), lactate (*n* = 4), fructose (*n* = 4), pyruvate (*n* = 4), galactose (*n* = 4), ß-hydroxybutyrate (*n* = 4), or insulin at 1.2 I.U. (*n* = 4). Saline (0.9%; *n* = 4) injected mice were used as the control group. Subsequently, glucose and lactate measures were taken at 30, 60, 90, and 120 min post-injection. This testing procedure was replicated for another saline group under two additional conditions in order to estimate the effect of stress. These groups were sampled every 5, rather than 30 min with half of them undergoing 15 min daily manipulation for 2 weeks (*n* = 4) while the other half did not (*n* = 4).

### Histology

Following testing, mice were lightly anesthetised using isoflurane and the electrodes gently removed for post-calibration. Following calibration, the mice received an i.p. injection of sodium pentobarbital (65 mg/kg; MERK, Kirkland, QC, Canada) before being transcardially perfused with a 4% paraformaldehyde/0.2% picric acid solution (PFA). Brains were then postfixed in the same PFA solution for 1 h at 4°C, then transferred to 10% sucrose overnight. The following day, the brains were frozen with CO_2_ and stored at −80°C for later cryostat sectioning at 14 μm. Electrochemical electrode placement within the primary motor cortex was confirmed with the use of the stereotaxic atlas of Franklin and Paxinos (2008).

### Statistical analysis

Glucose and lactate fluctuations observed in the periphery and extracellular space following various metabolic challenges were analyzed separately. A 2-h time frame was used because the observed metabolites usually returned to baseline or stabilized within this time.

### Relative measures

All data were standardized by dividing the readings, in nA, by their respective pre-event baseline and multiplying the result by 100 to obtain relative percentage change from baseline. For electrochemical electrode values, each baseline was individually calculated with an average of 10 s before injection and every 120 consecutive measurements were averaged in order to obtain a mean value every 2 min.

Glucose and lactate were analyzed separately with a between-within (split-plot) mixed ANOVA (α = 0.05), and the data obtained for each injection was compared to the saline injection group as well as baseline. Unless otherwise stated, the data met the respective assumptions of the analysis. If sphericity or homogeneity was violated, a Greenhouse-Geisser correction was applied. Due to the *a priori* decision and interest in differences across groups, *post-hoc* pairwise comparison tests (α = 0.05) were computed in order to compare the experimental groups to the saline controls as well as compare the experimental groups amongst themselves. All statistical analyses were performed using SPSS v. 23 (IBM).

## Results

Table 1 present a summary of blood and brain extracellular levels of glucose and lactate following systemic injections.

**Table 1:** **Summary of glucose and lactate trends following injections**.

	**Glucose**		**Lactate**	
**Injection**	**Brain**	**Blood**	**Brain**	**Blood**
Saline	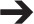 (Baseline)	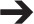 (Baseline)	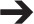 (Baseline)	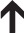 (~200%)
Glucose	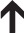 (~180%)	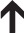 (~180%)	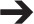 (Baseline)	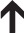 (~400%)
Fructose	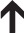 (~165%)	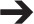 (Baseline)	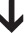 (~80%)	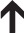 (~400%)
Lactate	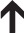 (~175%)	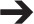 (Baseline)	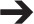 (Baseline)	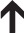 (~500%)
Pyruvate	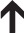 (~155%)	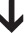 (~80%)	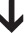 (~80%)	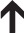 (~550%)
ß-Hydroxybutyrate	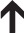 (~185%)	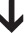 (~70%)	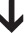 (~70%)	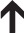 (~600%)
Galactose	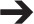 (Baseline)	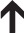 (~120%)	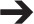 (Baseline)	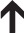 (~350%)
Insulin	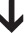 (~60%)	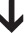 (~50%)	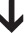 (~60%)	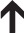 (~280%)

*Table 1. Illustration of the metabolite trend (glucose or lactate) in their respective compartments (blood and extracellular space of the brain) following the systemic injection of metabolic fuels. Arrows indicate whether the metabolite increased (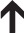), decreased (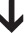) or remained at baseline (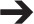). Values depict the approximate zenith or nadir achieved by these metabolites, in relation to their baseline (i.e., glucose decreased up to 80% its baseline value in the blood following a pyruvate injection)*.

### Effect of stress on peripheral values of glucose and lactate

**Figure 2:**
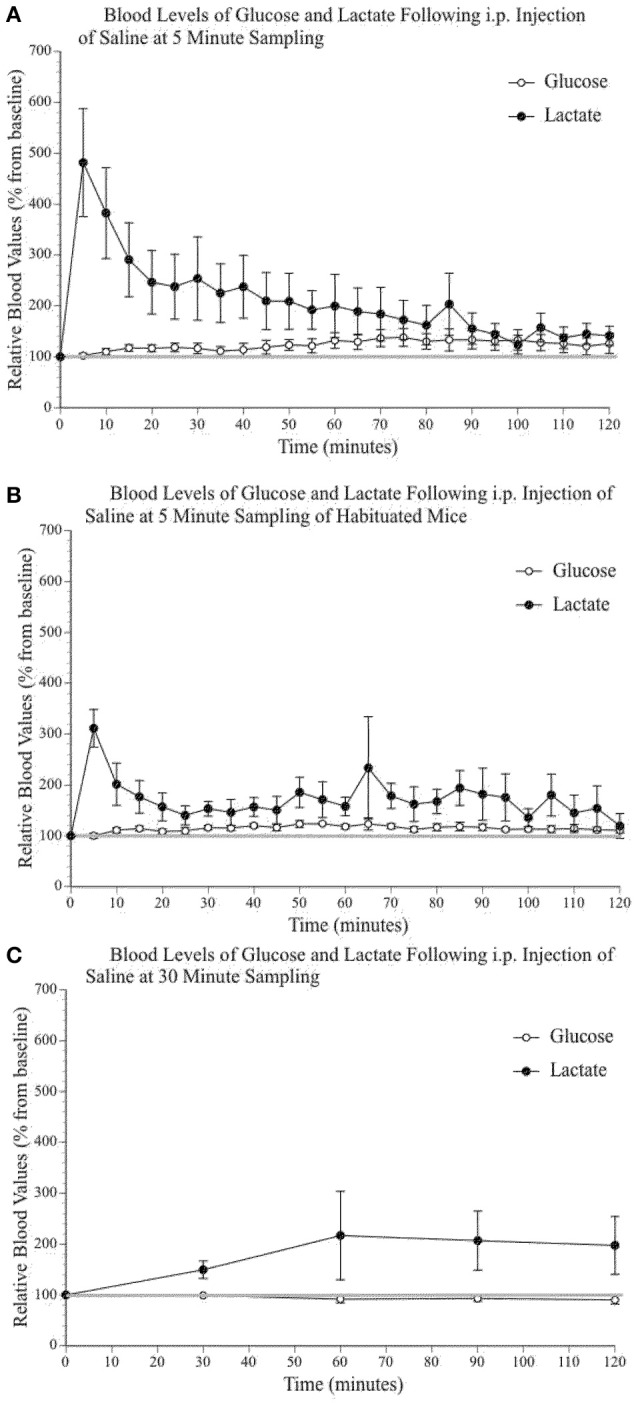
**Relative blood concentration of glucose and lactate following a 0.9% i.p. injection of saline (A)** at 5 min sampling interval in naïve mice (*n* = 4); **(B)** at 5 min sampling interval in habituated mice (*n* = 4); **(C)** and at 30 min sampling interval in naïve mice (*n* = 4). Values are expressed as a percent of their baseline value (taken prior to injection) and error bars represent the standard error of the mean.

It has been previously established that stress can increase systemic levels of glucose and lactate in a variety of species (Silbergeld, 1974; Wells et al., 1986; Nagamatsu et al., 1994; Hall and Van Ham, 1998; Adolphs et al., 1999; Rand et al., 2002; Gruber et al., 2010), including rodents (Barnett et al., 1960; Abel, 1994; Vachon and Moreau, 2001; Sim et al., 2010) and humans (Sharda et al., 1975; Munck et al., 1984; McCowen et al., 2001). Unsurprisingly, taking tail vein blood in awake mice can cause significant stress. Because we were testing the impact of substances that influence blood glucose and lactate, which can in turn have an impact on brain extracellular glucose and lactate levels, we used a sampling procedure that would minimize the effect of stress while still providing useful data. We tested a number of alternatives. Figure 2 illustrates the fluctuations of blood lactate and glucose following sampling conducted every 5 min without (2A) and with a 2-week habituation/handling period (2B). Figure 2C presents the results using a 30-min sampling interval. Split-plot mixed ANOVA did not reveal any significant effect of group for either blood glucose [*F*_(2, 9)_ 3,87, *p* = 0.061, Observed Power = 0.545] or lactate [*F*_(2, 9)_ 0,285, *p* = 0.759, Observed Power = 0.083] values. However, pairwise comparisons for glucose did indicate a significant between group difference when comparing the 5 min sampling paradigm to the 30 min sampling paradigm (*p* = 0.023) but not when comparing the 30 min sampling paradigm and the 5 min sampling paradigm with prior habituation (*p* = 0.107). Therefore, sampling every 30 min produced similar values as those obtained following a 2-week habituation period. Pairwise comparisons did not reveal statistically significant differences between the lactate levels of the different sampling paradigms. In summary, regular manipulation and habituation results in similar levels of physiological stress responses to the 30-min sampling paradigm. Thus, the following peripheral data were obtained with a 30-min sampling paradigm.

### Intraperitoneal injection of alternative fuels increases systemic lactate

**Figure 3:**
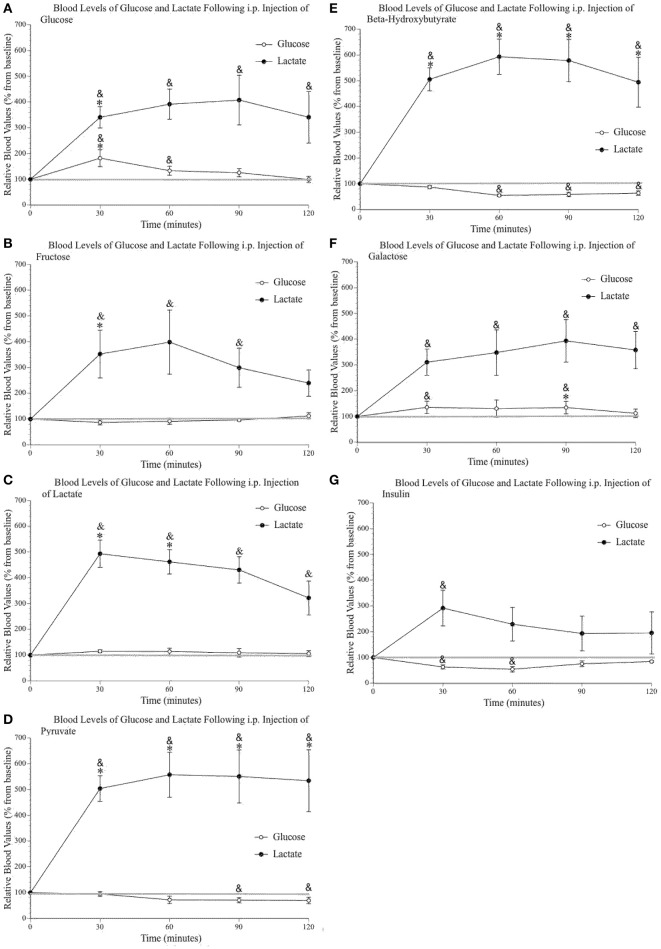
**Relative blood concentration of glucose and lactate following a 2 g/kg i.p. injection of (A)** glucose (*n* = 4); **(B)** fructose (*n* = 4); **(C)** lactate (*n* = 4); **(D)** pyruvate (*n* = 4); **(E)** β–hydroxybutyrate (*n* = 4); **(F)** galactose (*n* = 4). **(G)** Relative blood concentration of glucose and lactate following a 1.2 I.U. i.p. injection of insulin (*n* = 4). Values are expressed as a percent of their baseline value (taken prior to injection) and error bars represent the standard error of the mean. ^*^ = significant difference when compared to control (saline) values (*p* < 0.05) & = significant difference when compared to baseline (*p* < 0.05).

Figures 3A–E shows increased systemic lactate above saline values after each of the injected metabolites, while systemic glucose remained mostly unchanged (with the obvious exception of the glucose injection). The insulin injection (Figure 3G) reduced blood glucose values but did not change blood lactate levels except for the first 30-min sample, most likely a consequence of the i.p. injection and the related stress. The analysis of peripheral glucose and lactate indicated a significant effect of time [*F*_(4, 96)_ 4.59, *p* = 0.002, η^2^ = 0.160; *F*_(2.39, 57.45)_ 60.26, *p* < 0.000, η^2^ = 0.715] and injected substance [*F*_(7, 24)_ 4.10, *p* = 0.004, η^2^ = 0.545; *F*_(7, 24)_ 3.52, *p* = 0.010, η^2^ = 0.507], respectively. Pairwise comparisons were conducted to determine which group(s) significantly differed from control (saline) values as well as determine the precise times at which these values differed from both saline and baseline levels (as indicated on Figures 3 A–G). *Post-hoc* analyses demonstrated that the only injection capable of raising systemic glucose above saline values was, unsurprisingly, the glucose injection. Also, the injection of galactose slightly raised blood glucose above baseline values at 30 and 90 min post-injection. Pairwise comparisons confirmed that lactate, pyruvate, and ß-hydroxybutyrate injections increased blood lactate levels above saline values. The percent average blood lactate increase following the injection of glucose (*p* = 0.563), fructose (*p* = 0.290), galactose (*p* = 0.449), pyruvate (*p* = 0.267), and ß-hydroxybutyrate (*p* = 0.244) was not statistically different from that observed after a lactate injection. However, the injection of glucose, fructose and galactose produced smaller blood lactate increases and the comparison with saline injection values were only significant for glucose and fructose at the 30-min sample.

### Systemic injection of alternative fuels increases glucose and decreases lactate in the primary motor cortex extracellular space

**Figure 4:**
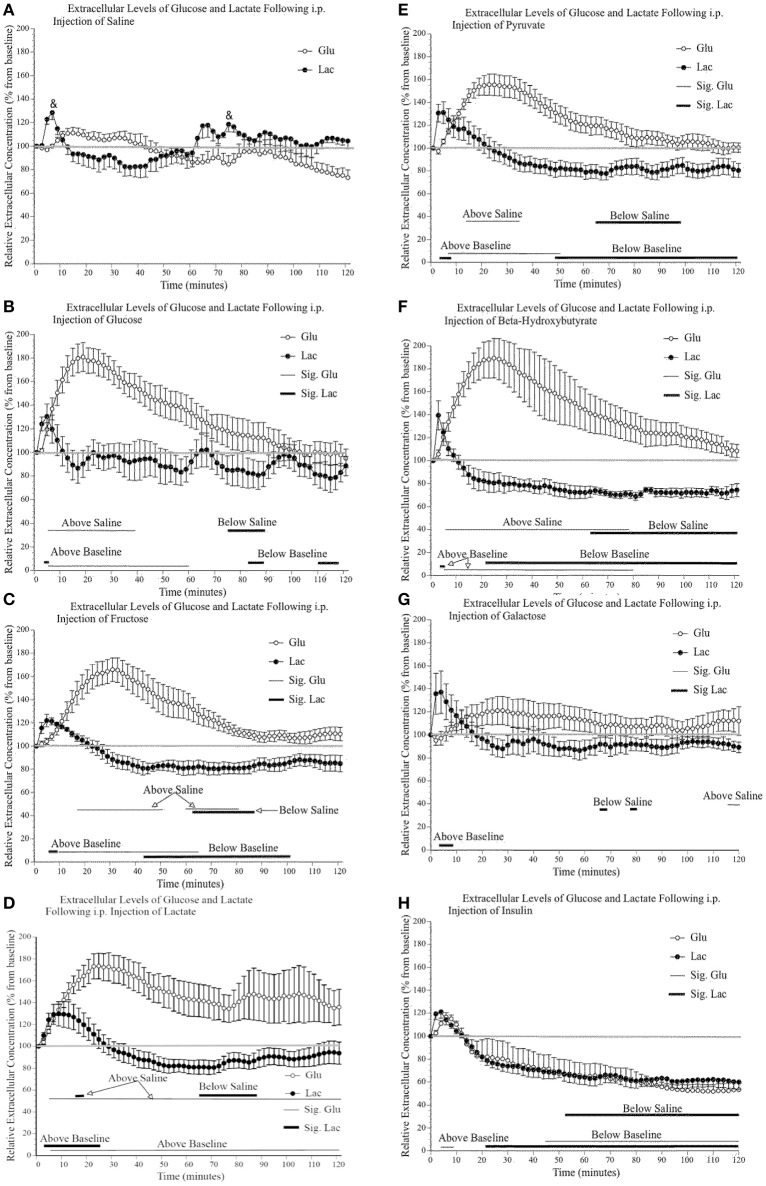
**Relative extracellular concentration of glucose and lactate following a (A)** 0.9% i.p. injection of saline (*n* = 4), a 2 g/kg i.p. injection of **(B)** glucose (*n* = 4); **(C)** fructose (*n* = 6); **(D)** lactate (*n* = 10); **(E)** pyruvate (*n* = 6); **(F)** β–hydroxybutyrate (*n* = 5); **(G)** galactose (*n* = 6), as well as **(H)** a 1.2 I.U. i.p. injection of insulin (*n* = 7). Values are expressed as a percent of their baseline value (taken prior to injection) and error bars represent the standard error of the mean. The thin horizontal lines below each graph represent significant tests for extracellular glucose values while the thicker horizontal line indicates significant tests for extracellular lactate values (*p* ≤ 0.05). Significant tests for comparisons between saline values are indicated separately from the comparison with baseline values.

Figure 4A shows the effect of a saline injection on extracellular glucose and lactate content in the primary motor cortex. Of note is the initial, significant transitory rise in extracellular lactate (30%), which returns to baseline 10–15 min post-injection. As this rise in extracellular lactate is consistently observed in all conditions immediately following injections, we suggest that it is a result of restraining of the mice and subsequent motor behavior. Indeed, injecting the mice require their restraint which results in struggling during the injection procedure. Immediately following the injection, mice typically engaged in enhanced grooming, and running behavior for approximately 10 min. Although we did not quantify motor activity during the post-injection period, the video recordings were annotated to show bouts of running or intense grooming. In general, the level of activity appeared higher in the saline group and was mostly similar across experimental groups except for mice receiving lactate, which were less active. Also, the changes in extracellular lactate and glucose associated with discrete motor activity were much less than the changes associated with the systemic injections and were not found to be a significant contributing factor in the present experiments. Finally, this small lactate increase associated with the initial injection occurred for all substances injected except for lactate; following a lactate injection, the injection-associated rise is greater (50%) and returns to baseline 30 min later. Additionally, toward the end of the 2-h testing period, there is a small steady decline in glucose level that is likely associated with the 4-h fasting.

Figures 4B–G show that the systemic injection of alternative fuels significantly increased extracellular glucose levels, beyond what was observed following a saline injection. Extracellular glucose but not lactate showed a significant effect of substance injected [glucose: *F*_(7, 40)_ 6.62, *p* < 0.000, η^2^ = 0.537; lactate: *F*_(7, 40)_ 2.09, *p* = 0.067, η^2^ = 0.268], and a significant effect of time [glucose: *F*_(59, 2360)_ 30.66, *p* < 0.000, η^2^ = 0.434; lactate: *F*_(59, 2360)_ 44.37, *p* < 0.000, η^2^ = 0.526], respectively. We were interested in examining the differences between saline and each substance as well the significance of the differences between the baseline values and values after injections. On each panel of Figure 4, we indicated the significance of pairwise comparison between the values obtained after the saline injection and those observed after each substance. The thin horizontal lines below each graph represent significant tests for extracellular glucose values while the thicker horizontal line indicates significant tests for extracellular lactate values.

When the effects of alternate fuels on extracellular glucose are examined, all substances except galactose produce a rise in extracellular glucose. This difference between galactose and other metabolic substrate will be addressed later in the discussion. Among these significant effects, there are similarities and differences in the slope of the initial increase in extracellular glucose and the time period during which extracellular glucose remains elevated. First, the extracellular glucose rates of rise following glucose, lactate and ß-hydroxybutyrate injections are very similar. Also, extracellular glucose rates are almost identical when comparing the effect of fructose and pyruvate injections. Possibly, the rapid rise in extracellular glucose observed after the systemic injection of glucose, lactate and ß-hydroxybutyrate is associated with their greater ability to cross the blood brain barrier or the ease with which they are systemically metabolized. In contrast, the delayed rise in extracellular glucose observed after the systemic injection of fructose, pyruvate and galactose may reflect the slower transport or the requirement of additional metabolic steps to transform fructose, pyruvate, and galactose into easily transportable substrates. Finally, we observed that the effect of the injection of lactate on extracellular glucose levels is much more variable than what was found for other injections. We could not identify any mediating variable for this variability in the present results.

The effects of alternate fuels on brain extracellular lactate present a different pattern altogether. First, the systemic injection of fructose, pyruvate, and ß-hydroxybutyrate all led to significant decreases in brain extracellular lactate. This decrease was absent following the systemic injection of glucose, lactate and galactose. We also noted that all systemic injections, including saline, briefly increased extracellular lactate levels but this increase was greater and more sustained after the systemic injection of lactate. We now turn to the effect of systemic insulin on extracellular glucose and lactate.

### Systemic insulin decreases glucose and lactate in the primary motor cortex extracellular space

The decrease in extracellular glucose following systemic insulin has been described previously (McCall et al., 1986) and is thought to be associated with the decreased blood glucose levels and the proportionally reduced gradient-dependent transport from blood to brain. Systemic insulin also reduced extracellular lactate at exactly the same rate and extent, as was extracellular glucose. The 40% decrease in extracellular lactate was greater than the one that followed the systemic injection of fructose (20%), pyruvate (20%), and ß-hydroxybutyrate (30%).

### Comparisons of trends between blood and brain extracellular levels of glucose and lactate following systemic injections

If the brain-blood barrier transport of glucose and lactate are solely taken into account, the brain extracellular levels of glucose and lactate should be proportional. For glucose, previous data has described a relation between blood and brain glucose that is usually described as brain glucose being 20–30% of blood glucose (Silver and Erecinska, 1994). This relation is maintained during fasting as well following insulin injections (Dunn-Meynell et al., 2009). In the present set of results, this relationship was observed following glucose and insulin systemic injections. However, the systemic injection of pyruvate and ß- hydroxybutyrate both led to a decrease in blood glucose and a rise in brain extracellular glucose. Conversely, the systemic injection of fructose and lactate did not raise blood glucose levels but led to an increase in brain glucose levels.

Finally, lactate transport is thought to be directional and dependent, among other factors, on concentration gradient across the blood-brain barrier (Figley, 2011). In the present experiments, only the systemic injection of lactate slightly increased brain extracellular lactate levels. Two other metabolic substrates, ß-hydroxybutyrate, and pyruvate, that increased blood lactate to similar levels as the systemic injection of lactate were associated with decreased brain extracellular lactate levels. Systemic injections of glucose, fructose and galactose all led to equivalent increased blood lactate levels but glucose and galactose did not produce lower brain extracellular lactate levels while fructose did. Together these observations suggest that blood and brain extracellular levels of glucose and lactate do not follow a simple distribution based on blood brain transport and concentration gradient between blood and brain.

In summary, the systemic injection of various metabolic substrates in awake and freely moving mice led to variations in brain extracellular lactate levels in the motor cortex (see Table 1 for a summary). These metabolic substrates were injected in quantities consistent with typical food ingestion or exercise and were not supra-physiological. Insulin injections did not impair the animals' movement and did not induce loss of consciousness. Although the experimental conditions are obviously artificial, we nonetheless devised test conditions that are relevant for normal physiological states. Finally, the animals across groups were comparable regarding their motor activity except the animals in the lactate group that tended to be less active. During their random bouts of activity, the extracellular glucose and lactate changes observed in the motor cortex of all mice were much smaller than that produced by the systemic injections. Also, the motor activity was randomly distributed throughout the test session with the exception of the period immediately following the systemic (i.p.) injection when the animals struggled briefly and moved about the cage after being released from the hold of the experimenter. Within the limits of the present experimental design, the present results provide new information on how metabolic fuels are transferred and used by the brain and also raise a number of questions that need to be addressed.

## Discussion

### Methodological considerations

Before we discuss the results of the present experiments, a number of aspects of the techniques used and the nature of the sampling have to be stated. First, we measured glucose and lactate extracellular levels in the motor cortex of freely moving mice. Although mice could move around the testing enclosure, the amount of activity was limited to a few bouts randomly spread out during the 2 h of observation. Second, the brain extracellular milieu is a site of bilateral exchange between the brain parenchyma and blood circulation. The electrochemical electrodes that we inserted in the motor cortex measure the content of glucose and lactate in that milieu (net flux) but give no information as to the directional flux (influx vs. efflux) of the metabolites. Thus, the brain extracellular milieu is like an antechamber where people go through to enter a room. Figuratively, our measures give information on the number of people transiting in the antechamber at any given time but no information on whether they are going into the room or leaving it.

We used percentage of change over baseline to present data to allow interindividual and group comparisons. In theory, the use of pre- and post-calibration could allow us to provide absolute values. However, a number of limitations make this difficult. First, pre-calibration of the electrodes provides the values that allow the direct conversion between current recorded and concentration of glucose or lactate. When the electrodes are inserted into the living brain, the physical electrode damages brain tissue and initiates the usual responses to insult, including inflammation and microglial activation. The adaptation period we used here (about 2 h) appears to lead to the stabilization of the recorded current at the electrode, which likely corresponds to the end of the acute response to tissue damage following electrode insertion. Following that period, the electrodes appear to react to brain activation and systemic injection rapidly and consistently. There is a small consistent and mostly linear decline in current over 24 h that is likely due to biofouling, the accumulation of biological material or cells on the electrode surface. Post-calibration, in theory, measures the electrode sensitivity at the end of the experiment. However, it has been a challenge to remove electrodes through the guide cannula holding the electrode in place without any damage to the membranes so as to provide reliable measurements across all animals.

The main concern with comparing percentage of change over baseline measures of glucose and lactate is their respective difference in absolute concentration in the brain. As calculated with the use of our electrode calibration, average baseline brain extracellular values of glucose and lactate were 1.4 ± 0.59 and 0.9 ± 0.56 mmol/L, respectively. Average blood values of glucose and lactate ranged from 8 to 10 mmol and 1.7 to 3.1 mmol/L, respectively. The observed brain extracellular glucose values fell within the expected 20% of blood concentration; however, brain extracellular lactate values were closer to 35–40% of blood values. Additionally, when glucose is metabolized through the glycolytic pathway, each molecule of glucose gives rise to two lactate molecules. As a consequence, analysis of relative changes of these metabolites in the brains' extracellular space, or the periphery, may lead to an overestimation of the absolute contribution these metabolites to brain energetics. For example, if blood lactate rises 400%, blood lactate concentration rises from 2.5 to 10 mmol/L. This near 400% rise in blood lactate is regularly observed in exercise studies, reaching values close to or above 10 mmol/L (Wasserman, 1967; Meek et al., 2009; Petrosino et al., 2016). In contrast, if blood glucose only rises 10%, blood concentration goes from 9 to 9.9 mmol/L. Thus, a 400% rise in lactate depicted in a graph may give the illusion that blood lactate levels greatly exceed that of glucose and these results interpreted as lactate having a greater relative contribution. With these caveats in mind, we now turn to possible hypotheses that can be used to account for our observations. These hypotheses are presented as possibilities and not as a conclusive interpretation of our results.

### Peripheral increases in lactate mediate some of the brain's use of systemic metabolic fuels

The primary finding culminating from this work is the observation of an uncoupling occurring between extracellular and blood values of our two metabolites of interest. Indeed, we observed a robust and long lasting increase of blood lactate following the systemic injection of glucose, fructose, lactate, pyruvate, and ß-hydroxybutyrate that was accompanied by maintained (ex: fructose) or even decreased (ex: ß-hydroxybutyrate) blood glucose levels. In contrast, brain extracellular levels of these two metabolites follow opposing trends with glucose increasing and lactate maintaining either a steady-state or decreasing. As such, individually, it would appear that these metabolites are uncoupled between the periphery and the brain.

Though these metabolites appear to be individually uncoupled (e.g.,: blood lactate levels not predicting brain lactate levels), the data suggest that they do not act independently of one another. A possible interpretation of the observed pattern of these metabolites and how they may influence each other would be that these metabolic substrates (except, of course, lactate) are first metabolized to lactate in the liver. The lactate produced by the liver then enters the brain and used either as a signaling molecule (Barros, 2013; Tang et al., 2014; Mosienko et al., 2015) or for oxidative metabolism (Waagepetersen et al., 1998a,b; Serres et al., 2003; Kasischke et al., 2004). Thus, metabolic substrates would first be transformed into lactate before the brain can use them. This view is supported by the negligible rise in blood glucose following the injection of most alternative fuels (except, of course, glucose) and the high rate of conversion of these substrates to lactate, including glucose (Kreisberg et al., 1970). The liver is capable of metabolizing various substrates to lactate. Indeed, pyruvate can be easily converted to lactate with the ubiquitous presence of LDH5 that favors the conversion of pyruvate to lactate (Wroblewski and Gregory, 1961; Fritz et al., 1969). Fructose can be converted to lactate through fructokinase, aldolase B and triokinase: enzymes that allow fructose to enter the glycolytic pathway as well as undergo gluconeogenesis (Exton and Park, 1968; Mayes, 1993). ß-hydroxybutyrate can be converted to aceto-acetyl CoA and enter the TCA cycle for oxidative metabolism (Bergman, 1971; Robinson and Williamson, 1980; Armulik et al., 2010) or, alternatively, be converted into acetone and enter the methylglyoxal pathway where it can ultimately be converted to lactate (Armulik et al., 2010; Glew, 2010). Thus, fructose, pyruvate and ß-hydroxybutyrate, after conversion to lactate, can enter the TCA cycle for oxidative metabolism without being first converted to glucose. Furthermore, some of these substrates have been shown to decrease the contribution of glucose carbon into CO_2_ (Rolleston and Newsholme, 1967; Ide et al., 1969) and hepatic glucose uptake (Davis et al., 1984; Larrabee, 1995). In addition to the liver's ability to convert most substrates to lactate, endothelial cells of blood vessels also exhibit a highly glycolytic nature that commonly metabolizes glucose, or other substrates, to lactate which hints toward favorable export of lactate to surrounding organs (Mann et al., 2003; De Bock et al., 2013; Eelen et al., 2015). The injection of glucose, fructose, lactate, pyruvate, and ß-hydroxybutyrate have been shown to raise blood lactate (Huckabee, 1958; Kaye et al., 1958; Sahebjami and Scalettar, 1971; Davis et al., 1984; Hallstrom et al., 1989; Pan et al., 2000; Abi-Saab et al., 2002; Revelly et al., 2005; Kohler et al., 2007) however this is the first demonstration and comparison of the similarity with which they seem to affect two major metabolic fuels (i.e., glucose and lactate).

Despite the liver's ability to convert these substances to lactate and lactate's ability to cross the blood-brain barrier and act as an alternative fuels, it is important to note that lactate, as a signaling molecule, may also regulate pathways involved in memory formation and (unsurprisingly) homeostatic metabolic properties of cells and organs (Barros, 2013; Boury-Jamot et al., 2016). In the brain, recent evidence has demonstrated presence of the *HCAR1* gene in neurons (Bergersen and Gjedde, 2012). In adipocytes, this gene codes for a G_*i*_-coupled lactate receptor capable of inhibiting adenylyl cyclase (Lovatt et al., 2012). Though the elucidation of the functional role of *HCAR1* is still lacking, the presence of such a receptor may further emphasize the role of lactate as a signaling molecule and its role in maintain metabolic homeostasis. Indeed, Barros (2013) states the limitations of pure neuronal glucose use and discusses the ways in which astrocytic lactate could be used. Of particular interest is their discussion of “metabolic recruitment by lactate” which illustrates how lactate could participate in maintaining metabolic homeostasis by means other than acting as an alternative fuel. For example, lactate could stimulate inhibitory GABAergic neurons (Shimizu et al., 2007) and promote lateral inhibition leading to increased metabolic availability of substrates to the remaining active neurons. Also, as demonstrated by Rovetto (Rovetto et al., 1975) and others (Gilbert et al., 2006; Shimizu et al., 2007; Ruusuvuori et al., 2010), lactate could increase the acidification of cells and therefore decrease their spontaneous activity. Interestingly, lactate may account for the observations that astrocytes decreased their use of glucose and glycogen in active regions of the brain (Pellerin and Magistretti, 1994; Loaiza et al., 2003; Porras et al., 2004, 2008; Bittner et al., 2011; Zhao et al., 2011) while decreasing their glucose use in less active brain regions (Sotelo-Hitschfeld et al., 2012), thus facilitating the diffusion of glucose from areas of low activity to areas of high activity.

In the next section, we explore three hypotheses that could explain the metabolic uncoupling observed in our data. We explore these options within a perspective of lactate as a metabolic fuel. However, it is crucial to remember that this is a limited perspective of how the brain may be adapting to these changes in the systemic availability of metabolites. Any changes or adaptations occurring could be the downstream consequence of the initial rise in extracellular lactate.

### Extracellular brain increases in glucose: gluconeogenic hypothesis

This hypothesis is based on the long-held belief that glucose is the preferred and principal brain fuel (Lipton and Robacker, 1983; Levine et al., 1998). The blood lactate increase following the systemic injection of glucose, fructose, lactate, pyruvate and ß-hydroxybutyrate is consistent with previous observations. However, explaining and understanding the increase in brain extracellular glucose following these systemic injections requires more thought. The hypothesis that lactate is a gluconeogenic precursor (Struck et al., 1965; Ross et al., 1967; Kreisberg et al., 1970; Bergman et al., 2000) and that the increase in brain extracellular glucose results from excess peripheral lactate being converted to glucose faces a number of objections. Although there has been indication of increased gluconeogenesis at the blood-brain barrier (Daneman et al., 2010), it is highly unlikely that this conversion would occur in endothelial cells at a rate significant enough to raise brain extracellular glucose levels by 30% as endothelial cells are highly glycolytic. Gluconeogenesis has been shown to occur in astrocytes (Wiesinger et al., 1997), however this would again imply that the lactate crosses the blood-brain barrier and is converted to glucose while no brain extracellular lactate increase is detected by the lactate biosensor. Indeed, our present data show that brain extracellular lactate decreases below baseline 20 to 40 min after the injection of fructose, pyruvate and ß-hydroxybutyrate. If we interpreted these observations using this hypothesis, it would imply that the rate of gluconeogenesis greatly exceeds the rate of lactate entry. This seems unlikely as astrocytes appear to favor the release of lactate and not glucose (Dringen et al., 1993). In addition, sustaining such a high rate of gluconeogenesis would be extremely costly as gluconeogenesis is an energy dependent process (Katz, 1985).

### Glucose sparing hypothesis

This hypothesis suggests that the increased brain extracellular glucose is the result of decreased glycolysis since, as we previously indicated, gluconeogenesis is a costly, energy-dependent process (Katz, 1985). In this hypothesis, astrocytes take up lactate and later shuttle it to surrounding neurons using the astrocytes' fine processes that ensheath synaptic sites. Similarly to the postulated ANLS, this alternative hypothesis would suggest that increased extracellular cerebral glucose is a consequence of increased lactate use and shuttle to neurons, thus limiting the need for glucose-based glycolysis and increasing lactate-based oxidative metabolism in neurons. Consequently, favoring lactate-based oxidative metabolism and decreasing glucose-based glycolysis would lead to the eventual accumulation of glucose in the brain extracellular space. *In vitro* work supports this notion as lactate has been shown to decrease glucose use in neuronal and astrocytic cell cultures (Tabernero et al., 1996; Schurr et al., 1999; Schurr and Gozal, 2011). Further evidence supporting the lactate-shuttling capabilities of astrocytes is the presence of lactate transporters that favor lactate-export (Mann et al., 2003; De Bock et al., 2013; Eelen et al., 2015) as well as the differential expression of LDHs in neurons and astrocytes. Neurons preferentially present with higher levels of LDH_1_, which favor the lactate to pyruvate reaction, while astrocytes preferentially present with higher levels of LDH_5_, which favor the pyruvate to lactate conversion (Venkov et al., 1976; Bittar et al., 1996; Laughton et al., 2000). In other words, astrocytes can convert the glycolytic by-product pyruvate to lactate and export it into the extracellular space.

However, the ability to export lactate is not sufficient to explain the rise and subsequent decrease in extracellular glucose. Indeed, if the brain extracellular glucose increase is the result of lack of use, transport kinetics and concentration gradient should counter that accumulation. However, as previously mentioned, lactate function in the brain is not limited to its metabolic properties but can also function as a signaling molecule capable of regulating pathways involved in memory formation and (unsurprisingly) homeostatic metabolic properties of cells and organs (Barros, 2013; Boury-Jamot et al., 2016). Enzyme assays in other tissues, such as the liver and kidney, have demonstrated the ability of acute and chronic lactate incubation to inhibit hexokinase (and other important glycolytic regulating enzymes) by regulating its association to mitochondria, thus rendering it more susceptible to inhibition (Leite et al., 2011), suggesting the possibility that high lactate transport could inhibit glucose transport. Additionally, heart perfusion techniques in rats demonstrated that one of the leading causes of decreased glycolysis in hypoxic hearts is a decrease in pH caused by the accumulation of lactate (Rovetto et al., 1975). Thus, large lactate accumulations appear capable of decreasing glycolytic glucose use in other organs. In contrast, another heart perfusion study in the rat demonstrated the ability of lactate to increase the translocation of GLUT1 to the plasma membrane (Medina et al., 2002). Interestingly, this increased transporter translocation did not lead to increased phosphorylation of glucose analogs but rather to a decrease of their phosphorylation. These results lead us to think that lactate may have similar effects in the mouse brain, resulting in decreased hexokinase activity and thus glucose use, but possibly increasing transport through increased GLUT1 externalization. In conclusion, the hypothesis that the brain extracellular glucose rise we observed after systemic injection of a number of metabolic substrates could be explained by the preferential use of lactate and the subsequent accumulation of glucose.

### Opportunistic hypothesis

A final consideration is the potential ability of the brain to use metabolic substrates that are in highest concentration in the blood, a capacity that may have a significant evolutionary advantage for bioenergetics. In this hypothesis, glucose is most commonly used as its blood concentration is usually significantly higher than other substrates. Indeed, all of the metabolic substances we injected systemically presumably raised their blood concentration and could cross the blood-brain barrier through GLUT1 or MCT1, albeit at variable rates (Gould et al., 1991; Halestrap and Price, 1999; Juel and Halestrap, 1999; Halestrap and Wilson, 2012). In addition, the brain possesses the necessary enzymes to metabolize these various substances (Venkov et al., 1976; Majumder and Eisenberg, 1977; Laughton et al., 2000; Ning et al., 2001; Funari et al., 2007; Diggle et al., 2010; McKenna, 2012). Finally, GLUT1 or MCT1 transporters and the enzymes associated with the transport of these metabolic substrates have been shown to be upregulated following chronic exposure to a specific metabolic substance (Adelman et al., 1966; Moore et al., 1976; Corring, 1980). Although it is not known whether the brain is using these substrates directly *in vivo*, some of the pre-requisite elements are present in the brain to allow this. Because we did not measure the blood content of each metabolic substance apart from glucose and lactate, it is difficult to evaluate the merit of this hypothesis to explain the present results.

### Galactose

Galactose was observed to significantly raise blood glucose and blood lactate but to a smaller degree than glucose. The liver is capable of metabolizing galactose using the Leloir Pathway that converts galactose to glucose-6-phosphate, the first metabolic product of glycolysis (Isselbacher, 1959; Cohn and Segal, 1973; Holden et al., 2003). In our experiments, there was almost no effect of systemic galactose on brain extracellular glucose and lactate. Based on the facilitated transport of glucose, we should have observed some increase in extracellular glucose with the caveat that galactose may have decreased brain glucose uptake by competing with glucose for its transport (Baur and Heldt, 1977) or inhibiting its phosphorylation by modifying hexokinase availability (Park and Johnson, 1955; Knull et al., 1973). Similarly, according to the ANLS corollary noted above, the modest increase in blood lactate could have led to an increased accumulation of brain extracellular glucose—an effect not observed in our experiments.

A likely explanation for the different systemic and extracellular responses to this particular substrate may lie in the rate of its metabolism. Fructose, pyruvate and β-hydroxybutyrate are all quickly metabolized or excreted. This is illustrated by studies evaluating blood content or carbon label excretion of various metabolites. These studies highlight the mouse's ability to excrete or metabolize over 80% of the injected metabolites within 2 h post-injection (Guggenheim and Mayer, 1952; Miller et al., 1952; Eiger et al., 1980; Cha et al., 2008; Wang et al., 2009). Particularly, fructose (4 g/kg) and β-hydroxybutyrate (12 mg) are capable of returning to blood baseline levels 30 min following their respective injections (Eiger et al., 1980; Cha et al., 2008). In contrast, within a 2 h window, one experiment showed that mice are only capable of metabolizing approximately 30% of injected galactose (Ning et al., 2001). In that experiment, the cell concentration of galactose was <10% of their blood concentration, indicating a slow uptake, likely a consequence of the competition between glucose and galactose transport. In addition to this slow rate of influx, it appears the metabolic rate of galactose is even slower indicating that its metabolism is not a function of its concentration-dependent transport but rather its limited enzymatic activity (Baur and Heldt, 1977). However, the galactose intermediate, galactose-1-phosphate, has been identified in the mouse brain, but galactose itself was not (Ning et al., 2001). These observations suggest that either the small amount of galactose that can cross the blood-brain barrier is quickly metabolized, or that the brain is capable of endogenously producing galactose-1-phosphate. Our observations that galactose produces little change in brain extracellular glucose and lactate could be solely ascribed to its limited rate of absorption. This conclusion has to be tempered with our observation that blood glucose and lactate were elevated following galactose injection. Perhaps blood levels of glucose and lactate have to reach a higher level to influence brain extracellular glucose and lactate.

### Insulin

The effect of systemic insulin injections is equally intriguing as it illustrates the impact of reducing blood glucose levels with only a small increase in blood lactate levels. The effect of insulin has to be contrasted with the effect of pyruvate and ß-hydroxybutyrate that both reduced blood glucose levels while increasing lactate levels. Following the systemic injection of pyruvate and ß-hydroxybutyrate, brain extracellular glucose levels are elevated in the face of decreasing blood glucose levels. Following an insulin systemic injection, there is a small transient increase in blood lactate levels accompanied by a concurrent decrease in blood glucose. Under these conditions, brain extracellular lactate and glucose monotonically decrease in unison. Together the effects of pyruvate and ß-hydroxybutyrate on blood and brain glucose suggest that high blood lactate levels can sustain high brain extracellular glucose levels even when blood glucose levels are low. Conversely, low blood glucose together with lower blood lactate levels cannot sustain high brain extracellular glucose levels and brain extracellular lactate cannot compensate for this decrease. In a preliminary experiment (data not shown) we observed that increasing blood lactate, 2 h after an insulin injection, led to an increase in brain extracellular glucose levels up to 50% above pre-insulin baseline suggesting that blood lactate can reverse the decrease in brain extracellular glucose levels produced by systemic insulin injections.

## Conclusion

We are aware that our observations, as they stand, cannot be used to establish the relative prominence of the hypotheses we entertained in the discussion because we did not measure glucose or lactate utilization within the different brain metabolic pathways nor did we measure the brain levels of most of the systemically injected substances. However, our results provide new and useful *in vivo* information obtained under physiological conditions. The inescapable fact that the brain eventually ceases to function in the complete absence of either glucose or oxygen has focussed the theories of neuroenergetics toward the oxidation of glucose as being the prime mechanism for energy acquisition of the mammalian brain. However, the ability of the brain to adapt to low glucose in insulin-injecting diabetes patients (Maran et al., 2000), to low oxygen in people at high altitude (Hochachka et al., 1996) and increased ketone body in diet (Roy et al., 2015; Murray et al., 2016) suggests that the brain makes use of more than one mechanism to acquire energy. These alternate mechanisms can compensate for decreased glucose and oxygen but can never completely replace oxygen- and glucose-dependent metabolic activities. This limitation suggests that some brain physiological activity is uniquely dependent on those particular sources of energy. One possible candidate would be the sodium/potassium pump that appears to be highly dependent, at least in part, on the glycolytic pathway (Mercer and Dunham, 1981; Lipton and Robacker, 1983; Raffin et al., 1992; Pellerin and Magistretti, 1996; Silver and Erecinska, 1997; Sepp et al., 2014). Our *in vivo* results are consistent with the hypothesis that alternate metabolic substrates can either be used by the brain and/or reduce the need for glucose under physiological conditions.

## Author contributions

AB planned and performed experiments, completed the data analysis and wrote the manuscript. JL, JC, and TY performed and analyzed preliminary experiments and helped refine experimental protocols and data analysis. CM planned experiments and contributed to the data analysis and writing of manuscript.

## Funding

CM received a grant from the Natural Sciences and Engineering Council of Canada, and an equipment grant from the Canadian Foundation for Innovation and the Ontario Research Fund—Research Infrastructure program. We also acknowledge the support of the Faculty of Social Sciences of the University of Ottawa. JC and TY received an Undergraduate Summer Research Fellowship from the Natural Sciences and Engineering Council of Canada.

### Conflict of interest statement

The authors declare that the research was conducted in the absence of any commercial or financial relationships that could be construed as a potential conflict of interest.
